# Fabrication of Thermochromic Membrane and Its Characteristics for Fever Detection

**DOI:** 10.3390/ma14133460

**Published:** 2021-06-22

**Authors:** Hyeon Seop Jeon, Jeong Hwa Kim, Martin B. G. Jun, Young Hun Jeong

**Affiliations:** 1 Department of Mechanical Engineering, Graduate School, Kyungpook National University, Daegu 41566, Korea; hsjeon1994@gmail.com (H.S.J.); qhfekrn89@gmail.com (J.H.K.); 2 School of Mechanical Engineering, Purdue University, West Lafayette, IN 47907, USA; mbgjun@purdue.edu; 3 School of Mechanical Engineering, Kyungpook National University, Daegu 41566, Korea

**Keywords:** thermochromic pigment, 3D printing, temperature, fever monitoring

## Abstract

Body temperature is an important indicator of the health status of the human body. Thus, numerous studies have been conducted in various fields to measure body temperature. In this study, a biocompatible thermochromic membrane that changes its color when the temperature becomes higher than the transition temperature for thermochromism was fabricated using an extrusion-based three-dimensional printing process. The printing material was prepared by mixing a thermochromic pigment and a thermoplastic polymer in various ratios. The effects of mixing ratio on the various properties of the fabricated membranes were experimentally investigated. It is presented that the fabricated lattice membrane had excellent thermochromic reaction, which was experimentally evaluated using a measurement of color brightness. The pigment content affected the diameter and surface morphology of the printed filament. The elastic modulus decreased, and thermochromic response became faster as the pigment concentration increased. Subsequently, a patch for fever detection was developed and then attached to the skin to demonstrate its color change according to body temperature. Results show that the fabricated thermochromic patch could be successfully applied to fever detection.

## 1. Introduction

Research on human body-temperature measurement has been conducted in various fields, such as medicine, biotechnology, electronics, and mechanical engineering. Given the worldwide epidemic of infectious diseases, temperature measurement methods have been receiving a large amount of attention [1]. Body temperature, which is one of the vital signs of the human body, is an important index for diagnosing a person’s health condition [2]. Through body-temperature measurement, the onset and progress of infectious and noninfectious diseases, such as cardiac arrest and stroke, can be observed [3].

Body temperature is mainly obtained by using a contact or non-contact thermometer. Contact thermometers make contact with measurement areas, such as the armpit, oral cavity, and rectum. These thermometers are widely used in home and medical settings. Non-contact thermometers, also called infrared thermometers, work by detecting the infrared radiation spectrum emitted from the human skin. Infrared thermometers obtain measurements from the forehead and eardrum [4,5]. The measurement methods introduced above commonly require nursing personnel to perform body-temperature measurements periodically. However, if the patient develops a fever between the measurement cycles and does not take appropriate measures, they may experience physical abnormalities due to high fever. Those who have difficulty in communication (infants or patients with disabilities) may be seriously affected by these problems.

Continuous body-temperature measurement is helpful for the early detection of fever in high-risk patients. With early detection, patients can receive appropriate medical care. Particularly, the patient’s body temperature must be continuously monitored in an intensive care clinical practice [6]. Lin et al. developed a continuous body-temperature measurement system based on a thermal camera [7]. Sun et al. continuously checked human vital signs, including body temperature, using smart vital-sign monitoring equipment [8]. Yamasue et al. attempted to measure core body temperature wirelessly using an ingestible capsule-type sensor [9]. However, the above studies require expensive equipment, which is likely to pose a significant problem in small hospitals and developing countries [10].

Thermochromism is a property in which objects change colors in response to temperature; therefore, it is a valuable candidate for temperature monitoring. Thermochromic material changes its color or typically becomes transparent when the temperature becomes higher than the transition temperature for thermochromism. Many studies on thermochromic polymers and pigments have been conducted. Seeboth et al. developed a polymer gel embedded with a thermochromic dye and showed that it can be applied to a wide range of fields, such as sensors, displays [11], and medical and food fields [12]. Kim et al. fabricated a thermochromic polymer film embedded with organogel fiber [13]. Kulčar et al. studied the colorimetric properties of thermochromic inks [14].

In this study, a thermochromic membrane, which responds to temperature, was fabricated using an extrusion-based three-dimensional (3D) printing process. The printing material was prepared by mixing a thermochromic pigment and a biocompatible polymer. Then, various properties, such as surface morphology, filament diameter, and thermochromism, and the mechanical properties of the membrane fabricated with different pigment concentrations were experimentally investigated. Results demonstrated that the membrane could be successfully used as a fever detection aid for patients.

## 2. Materials and Methods

### 2.1. Materials and Experimental Equipment

**Figure 1:**
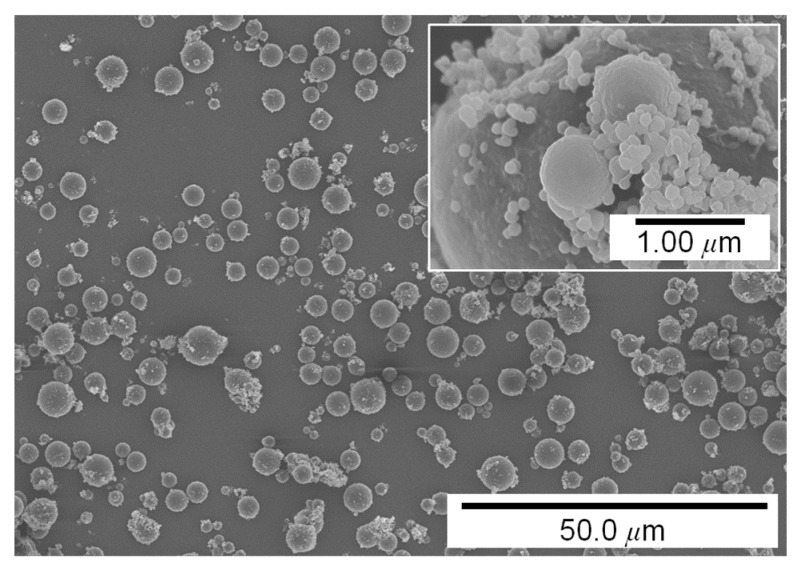
Thermochromic pigment powders.

In this study, a powder–polymer mixture was prepared with a biocompatible polymer polycaprolactone (PCL, Mw: 43,000–50,000, Polyscience, Warrington, PA, USA) and a fluorane leuco dye-based thermochromic pigment (Zion, Nano I&C, Asan, Korea) [15]. The particle size of the pigment varied from approximately 0.1 to 5 μm, as shown in Figure 1. Three types of mixtures were prepared with different mixing ratios of the pigment to PCL, that is, 5, 10, and 15 wt.%. The mixtures were made by stirring the mixture of pigment powders and the molten PCL for 45 min on a hot plate at 150 °C. Then, it was cooled at room temperature and finally cut into powder–polymer mixture scraps with a pair of scissors.

**Figure 2:**
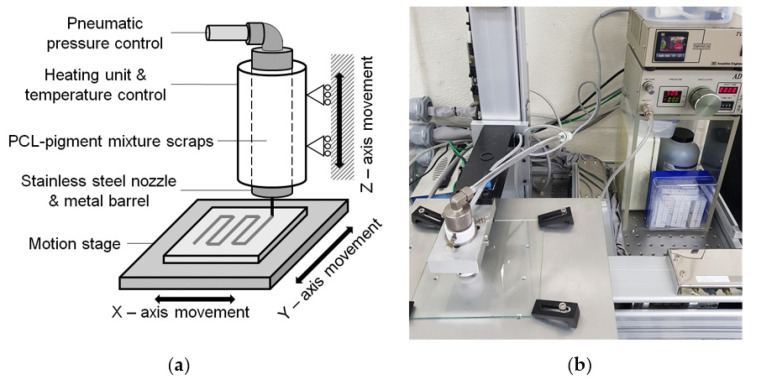
Experimental setups for extrusion-based 3D printing: (**a**) schematic diagram and (**b**) actual apparatus.

The thermochromic membrane was fabricated using an extrusion-based 3D printing setup, which consisted of a three-axis linear motion stage equipped with servomotors, a CNC controller (Clipper, Delta Tau, Los Angeles, CA, USA) for stage motion control, a temperature control (TCD-200EX, Iwashita, Kitakyushu, Japan), a pneumatic pressure control (AD 3000C, Iwashita, Kitakyushu, Japan), a metal nozzle with an inner diameter of 310 μm, and a metal barrel. Figure 2 shows a schematic of the extrusion-based 3D printing setup and the actual one used in this study.

### 2.2. Fabrication of Lattice-Shaped Structure

**Figure 3:**
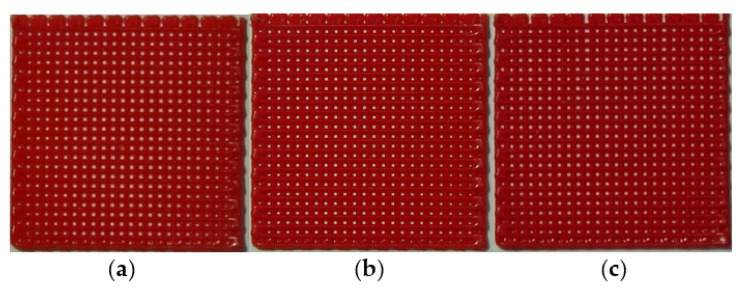
Fabricated lattice-patterned membranes with different pigment concentrations: (**a**) 5, (**b**) 10, and (**c**) 15 wt.%.

In this study, a lattice-pattern membrane with a size of 16 × 16 mm^2^ (square) was fabricated because the lattice pattern had a large surface area to volume ratio, and it was susceptible to temperature change. The grid size of the lattice pattern was 1 mm. The printing conditions are summarized in Table 1. Figure 3 shows the fabricated membranes with different pigment concentrations. A clear difference in color and shape among the samples was difficult to find.

### 2.3. Morphology Observation and Tensile Tests

**Figure 4:**
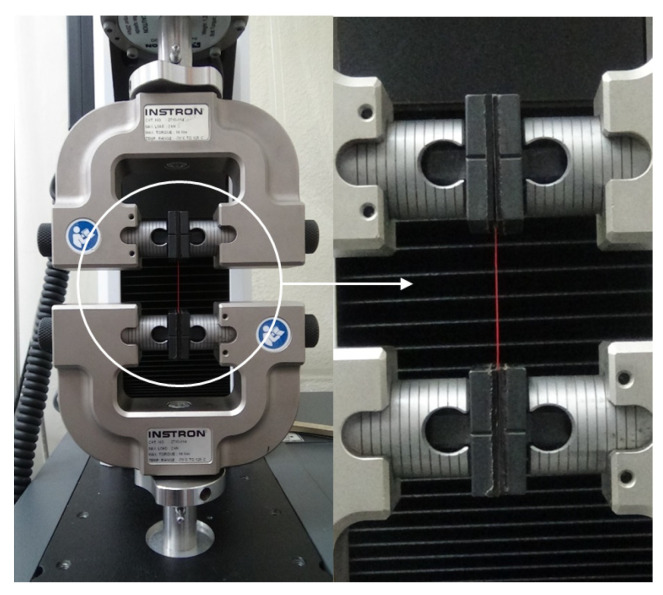
Experimental setup and a specimen for tensile test.

The morphology of the printed filaments was observed using a field-emission scanning electron microscope (SU8220, Hitachi, Tokyo, Japan) after palladium sputtering. ImageJ software was used for measuring the filament diameter from scanning electron microscope images. Tensile tests were conducted to determine the mechanical properties of the printed filaments by using a universal testing machine (UTM; 3400 series, Instron, Norwood, MA, USA). The specimens for tensile tests were single filaments printed under the conditions shown in Table 1. The gage distance of the specimen and the crosshead speed in the tensile tests were 30 mm and 5 mm/min, respectively [16]. The tensile test for each pigment concentration condition was repeated 10 times with fresh specimens. Figure 4 shows a specimen mounted on the UTM for the tensile test.

### 2.4. Color Changing at Transition Temperature and Image Processing

The fabricated membrane was placed in distilled water at 37.5 °C to observe the color change. The temperature of the water was kept constant by using a hot plate with a thermocouple probe. Eight specimens for each concentration were used. The color change in each sample was recorded using a digital camera with a resolution of 1280 × 720 and a frame rate of 30 frames per second.

**Figure 5:**
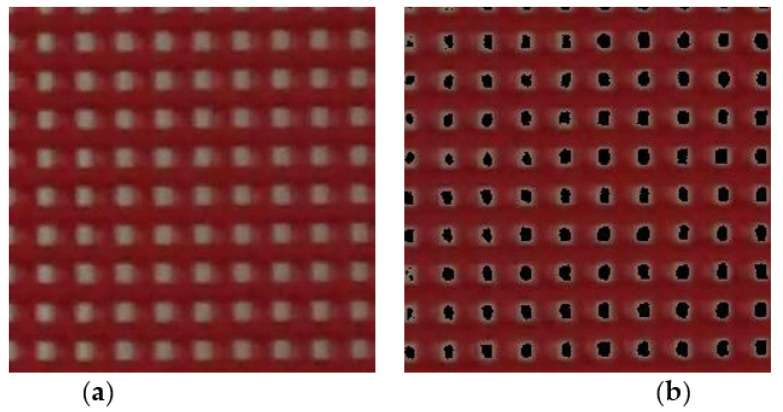
Image processing for background area removal: (**a**) before and (**b**) after background removal.

To quantify the color change in the membrane with respect to time, each image frame of the captured image sequence was processed and analyzed using MATLAB(R2020b) software (MathWorks, Natick, MA, USA). First, the red, green, and blue (RGB) values of each pixel were obtained for each image frame. While the color of the membrane changed from red to white at the transition temperature, the R value remained relatively constant, and the G and B values changed remarkably (Figure S1). From this phenomenon, the color of each pixel was identified, and the background area was detected and removed. A captured membrane image frame before removing the background area information is shown in Figure 5a. However, Figure 5b shows the image frame without background area information, which is represented with a black color in the figure.

In this study, color change was quantified using the brightness information of the color. Color brightness was defined as the arithmetic average of the R, G, and B values of each pixel [17]. Then, the average brightness of a membrane was obtained from the brightness information of all the pixels of a membrane.

## 3. Results and Discussion 

### 3.1. Influence of Pigment Concentration on Filament Diameter and Surface Quality

**Figure 6:**
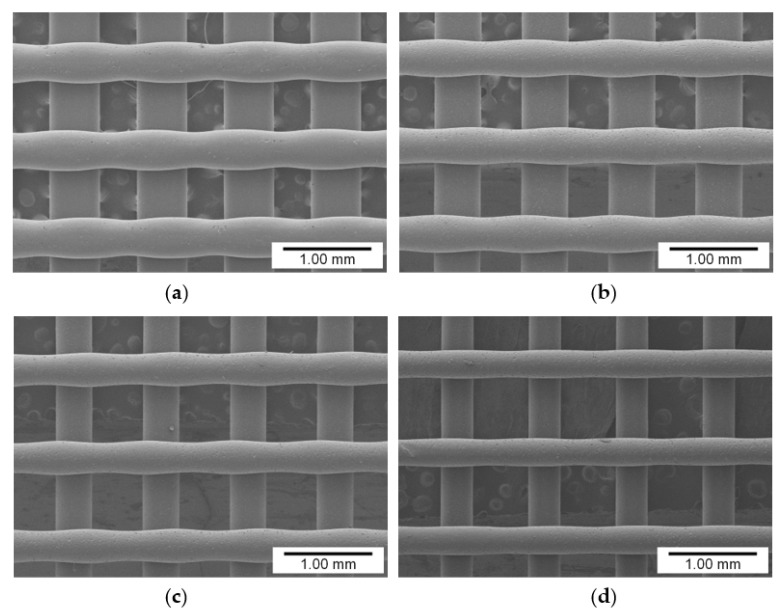
Lattice patterns of the membranes fabricated with different pigment concentrations: (**a**) 0, (**b**) 5, (**c**) 10, and (**d**) 15 wt.%.

**Figure 7:**
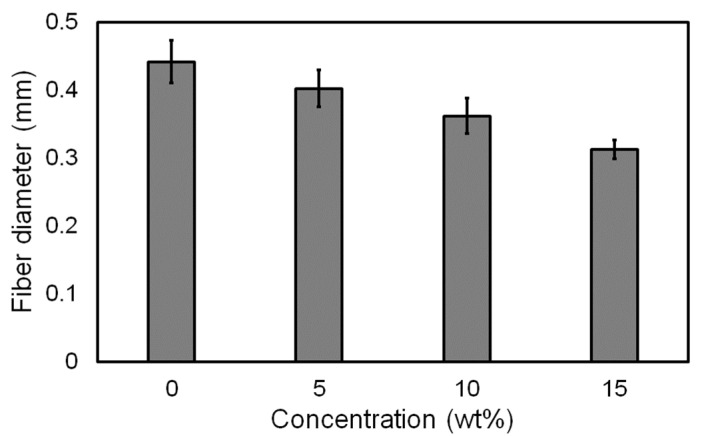
Fiber diameter according to pigment concentration.

Figure 6 shows the lattice patterns of the membranes that were fabricated using PCL–pigment mixtures with different pigment concentrations under the conditions mentioned in Table 1. The filament diameter became smaller as the pigment concentration increased because the viscosity of the molten mixture increased with the increase in the amount of added pigment [18]. The fluctuation of filament diameter could be found in all the samples, and the fluctuation resulted from the flattening effect (Figure S2). Filament diameter is compared to pigment concentration in Figure 7. The diameter was measured for the upper-layer filaments (i.e., horizontal filaments in Figure 6). As shown in the figure, the filament diameter decreased by approximately 40 μm on average as the concentration increased by 5 wt.%.

**Figure 8:**
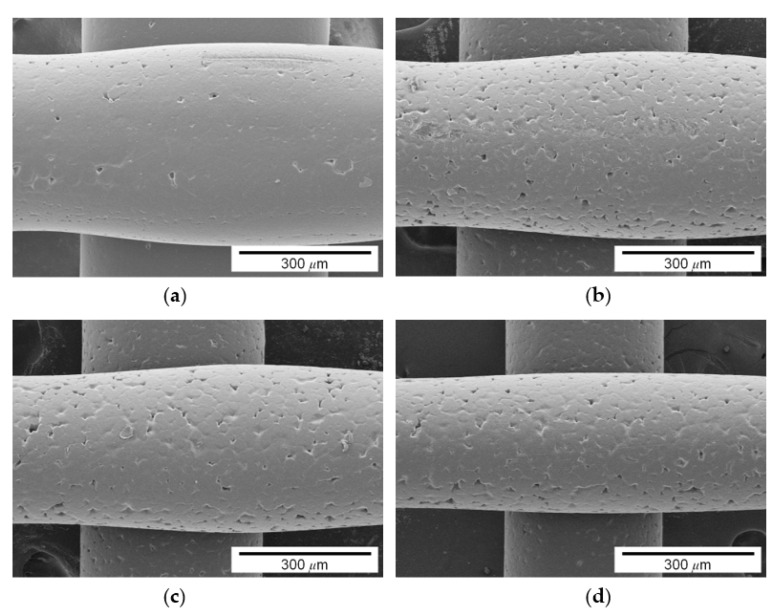
Surface morphology of the filaments with different pigment concentrations: (**a**) 0, (**b**) 5, (**c**) 10, and (**d**) 15 wt.%.

Pigment concentration also affects the surface quality of the printing, as shown in Figure 8. While the surface of the printing made of pure PCL (i.e., 0 wt.% of pigment) was relatively smooth, the surfaces of printing made of 5, 10, and 15 wt.% pigment–PCL mixtures had many pores. As shown in the figures, the addition of pigment powders induced the aggregation of molten PCL–pigment powders. 

### 3.2. Influence of Pigment Concentration on Mechanical Properties

**Figure 9:**
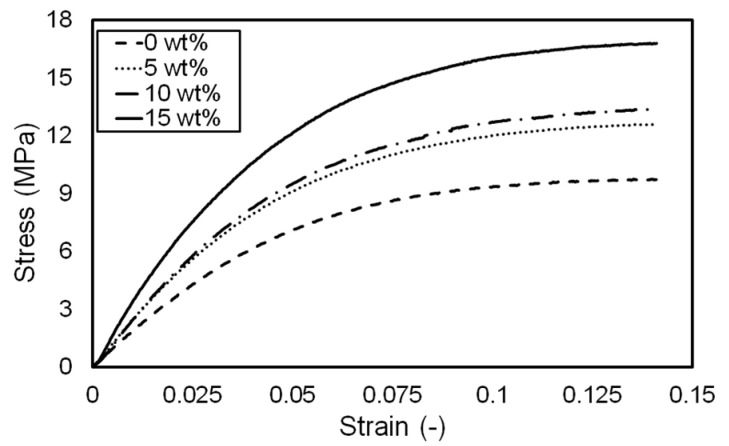
Stress–strain curves with respect to pigment concentration.

**Figure 10:**
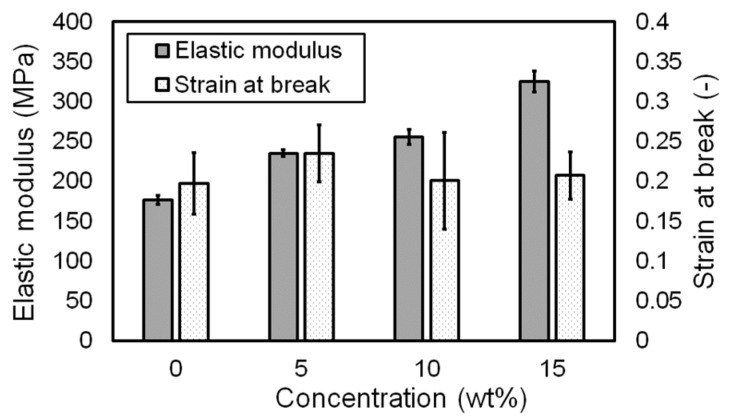
Elastic modulus and strain at break with respect to pigment concentration.

The influence of pigment concentration on the material properties of the printing was investigated. Figure 9 shows the stress–strain curves obtained from the tensile tests. As the pigment concentration increased from 0 to 15 wt.%, the printing became stronger because the pigment powders acted as structural reinforcement [19]. However, the stress–strain curves of 5 and 10 wt.% specimens did not show significant differences. The elastic moduli of the specimens with different pigment concentrations are shown in Figure 10. The elastic moduli were calculated in the strain range between 0.005 and 0.015 of stress–strain curves shown in Figure 9. Similar to stress–strain curves, the elastic modulus increased with the increase in pigment concentration. In addition, the strain at break (or fracture strain) of each specimen was investigated, as shown in Figure 10. All specimens suffered from fracture at a strain between 15 and 25%. However, any trend or influence of pigment concentration on strain at break could not be found in contrast to the elastic modulus. Moreover, given that the fracture strain had a large deviation, no meaningful conclusions could be drawn. The obtained elastic modulus and strain at break are summarized in Table 2. The standard deviations of the elastic modulus and strain at break were less than 4 and 30%, respectively.

**Figure 11:**
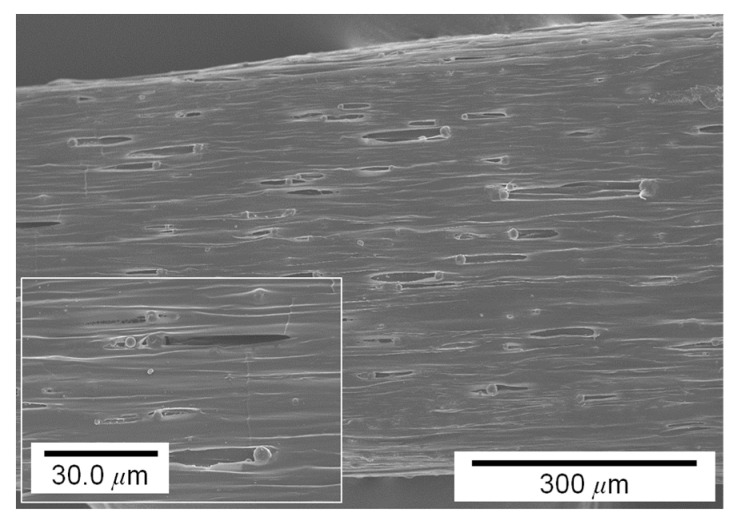
Surface morphology of the area close to the fracture of a test specimen after tensile test.

Figure 11 shows the surface of the area close to the fracture of a test specimen with a pigment concentration of 5 wt.%. Numerous grains can be found along the tensile direction. In particular, many groove defects were formed from powder–matrix interfaces when they suffered from stretching during the tensile test. These results reveal that the non-melt pigment powders were mixed with molten PCL, and the reinforcement powders provoked cavities under a large elongation condition. 

### 3.3. Influence of Pigment Concentration on Change in Color at Target Temperature

**Figure 12:**
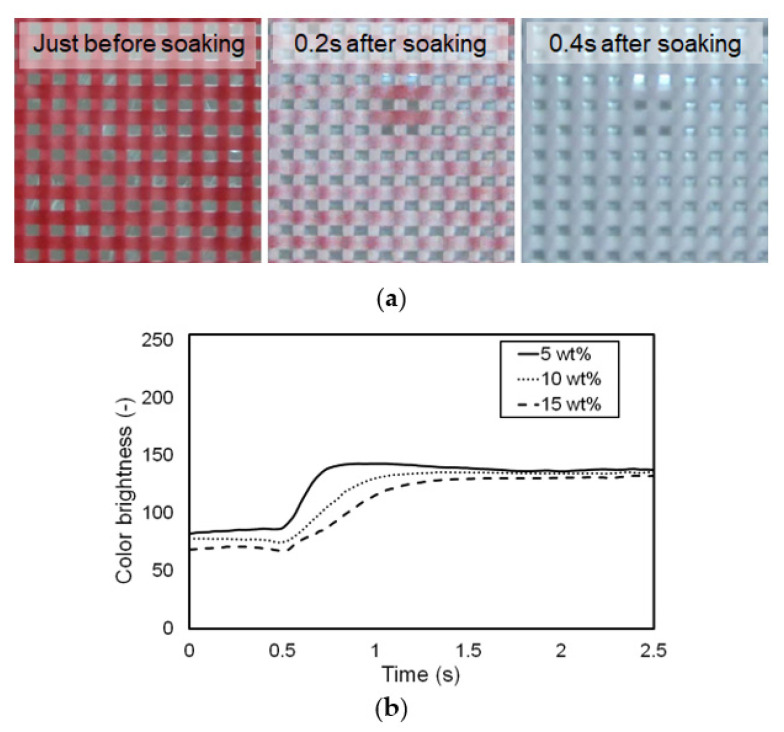
Color change in the fabricated membranes in warm water: (**a**) color change in a membrane with a pigment concentration of 5 wt.%; (**b**) color brightness change behavior of the membranes with three different pigment concentrations when they were soaked from air in warm distilled water (37.5 °C) at an instant of 0.5 s.

A membrane with a pigment concentration of 5 wt.% showed color change from red to white when it was soaked in distilled water at 37.5 °C, as demonstrated in Figure 12a. The membrane color successfully changed over its entire area when the membrane temperature reached the transition temperature of the pigment. Figure 12b shows the color brightness change (equivalently, color change) behavior of the membranes with different pigment concentrations. The membranes were soaked in distilled water (37.5 °C) at an instant of 0.5 s. As shown in Figure 12b, the color brightness change in all types of membranes with different pigment concentrations was completed earlier than approximately 1.0 s after the temperature change (i.e., soaking) and was maintained. In particular, the lower the pigment concentration, the shorter the required time for the settlement of brightness change.

**Figure 13:**
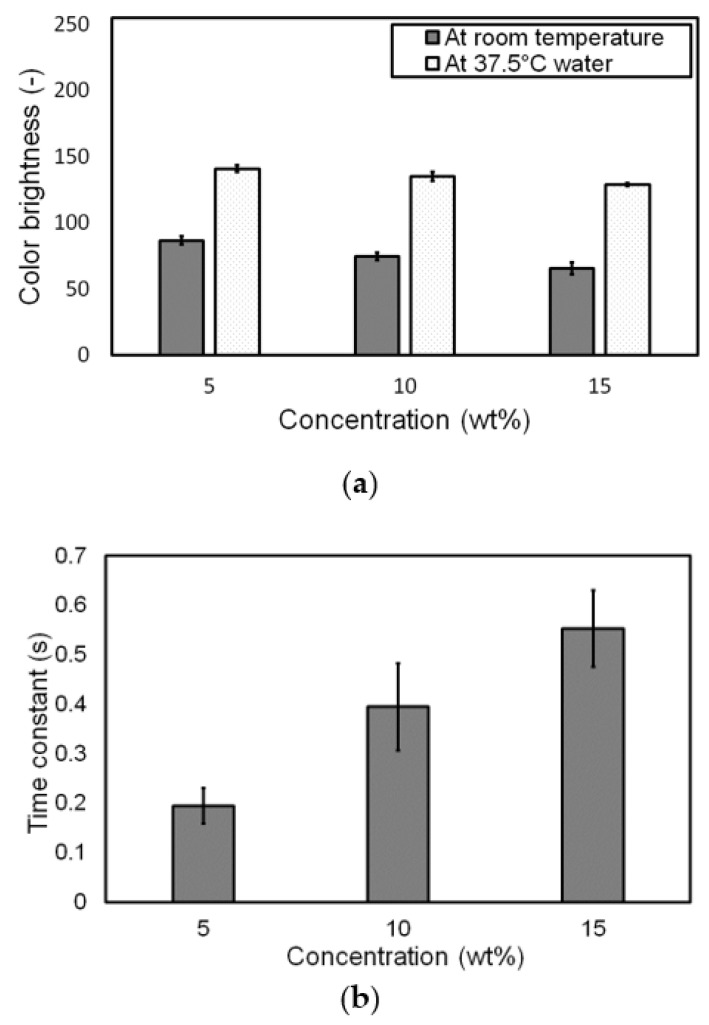
Analysis of color change behavior of the membranes with three different pigment concentrations when they were soaked from air into warm distilled water (37.5 °C): (**a**) change in color brightness and (**b**) time constant of color change.

Figure 13a shows the comparison results of the color brightness of all types of membranes with different concentrations between room temperature and 37.5 °C. At room temperature, the color brightness of a membrane with a high concentration decreased due to the large amount of pigment powder, which made the membrane’s red color dark. Meanwhile, the difference in the color brightness of the membranes at a temperature of 37.5 °C was reduced because the powder color became a bright one, that is, close to white. The obtained color brightness values are listed in Table 3; their standard deviations were less than 7%, indicating that the fabricated membranes had consistent thermochromic properties.

The response time of color change to temperature was investigated. As shown in Figure 12, the time required to change color at 37.5 °C with respect to pigment concentration varied. The higher the pigment concentration of the membrane, the longer the required time to change its color. To identify the effect of pigment concentration on response time, the time constant of color change was investigated. The time constant, which is typically defined as the time taken for a specified parameter to vary by a factor of approximately 0.6321 corresponding to 1-e^−1^ [20], was used to compare the response time of the membranes with different pigment concentrations. Figure 13b shows the time constant of color change with respect to the pigment concentration. The time constant was the smallest in a 5 wt.% pigment concentration, indicating the fastest response to temperature change. The time constant tended to increase with the increase in pigment concentration, despite a significant standard deviation because a large amount of pigment, which corresponded to high concentration, needed additional heat energy to provoke the thermochromic and endothermic processes. A large standard deviation may have been induced by insufficient mixing quality and experimental error in soaking the samples into the 37.5 °C water, even though the mixing quality was preliminarily verified by the almost identical RGB values at arbitrarily selected spots on a membrane (Figure S3 and Table S1). The obtained time constant with respect to pigment concentration is summarized in Table 3. 

### 3.4. Fabrication of Thermochromic Patch for Fever Detecting

**Figure 14:**
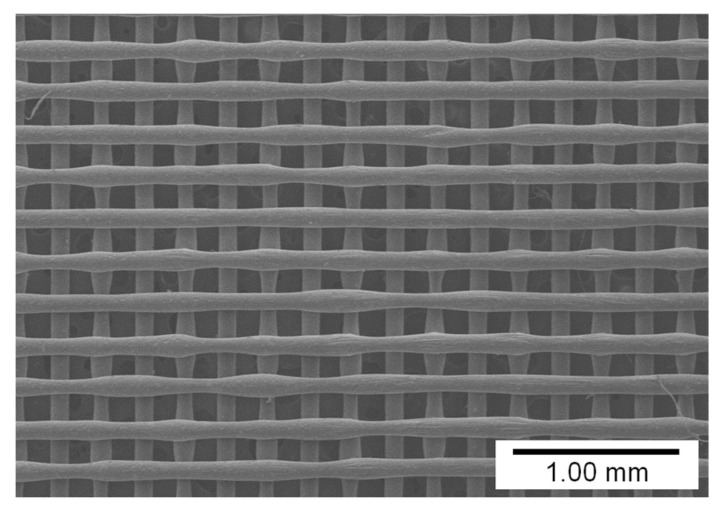
Fabricated skin patch for fever detection.

A thermochromic skin patch to detect fever was fabricated. The patch was made of PCL with 5 wt.% pigment concentration, which had the best compliance and responsibility to temperature changes. To improve the compliance for easy and solid adhesion to skin, a finer filament had to be extruded. Therefore, the previous nozzle with a diameter of 0.31 mm was exchanged with a nozzle with a diameter of 0.13 mm. In accordance with nozzle diameter, the tip-to-collector distance was reduced to 0.12 mm. In addition, pressure and feedrate, corresponding to printing speed, were modified. The printing conditions are summarized in Table 4. Figure 14 shows the fabricated skin patch. The patch size was about 16 × 16 mm^2^ (square), and the extruded filament diameter was approximately 0.12 mm.

**Figure 15:**
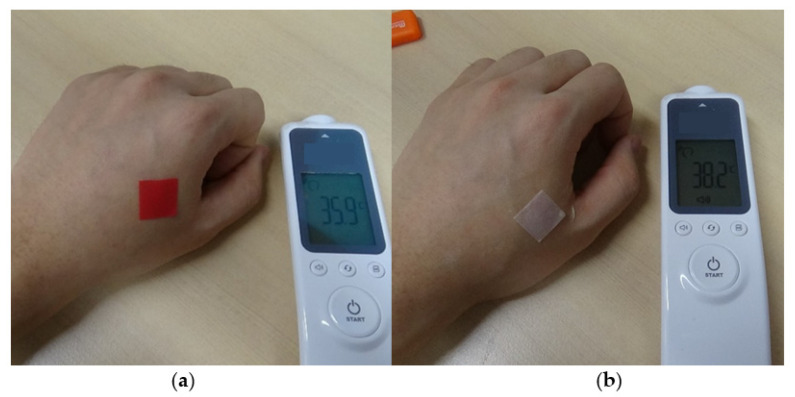
Patch attached to the skin on the back of the hand with different temperatures: (**a**) normal temperature condition and (**b**) fever condition.

To justify the feasibility of fever detection, the patch was attached to the skin on the back of the hand using a glycerin-based viscous gel (Eloglyn R995, LG Household & Health Care). Figure 15 shows the patches attached to the skin on the back of the hand with different temperatures, which correspond to normal and fever conditions. The color of the patch attached to the back of the hand at normal body temperature (35.9 °C) was red. However, the color of the patch under fever condition (38.2 °C) turned white. The time constant of color change was about 2.0 s, which was different from the time constant when a sample was submerged in the 37.5 °C water (Figure 13b). The difference in time constant resulted from the different heat transfer conditions. During the experiments, a side of a patch was attached to the skin, and the other side was open to the air with a temperature of 20 °C. These results confirm that the fabricated patch could successfully detect body fever.

## 4. Conclusions

In this study, a lattice-patterned thermochromic membrane that changes color in response to temperature change was fabricated through an extrusion-based 3D printing of a PCL–thermochromic pigment mixture. The effects of pigment concentration on various properties, such as filament diameter, surface morphology, elastic modulus, and thermochromic responses, were experimentally investigated. The filament diameter decreased as the pigment concentration increased under the same 3D printing condition because the viscosity of the molten polymer–pigment powder mixture increased with the increase in pigment concentration. In terms of mechanical properties, the elastic modulus increased with the increase in pigment concentration because the pigment powders acted as reinforcement. In this study, color brightness was used to quantify color change. The lower the pigment concentration of the mixture, the shorter the time needed to change color. A skin patch for a feasibility test of fever detection was fabricated. The test results demonstrated that the patch could successfully respond to human body fever or temperature change.

## Figures and Tables

**Table 1:** Printing conditions for lattice-pattern membranes with different pigment concentrations.

Parameter (Unit)	Value			
Concentration (wt.%)	0	5	10	15
Barrel temperature (°C)	100			
Ambient temperature (°C)	20			
Pressure (kPa)	550			
Nozzle inner diameter (mm)	0.310			
Extrusion speed (mL/h)	0.316	0.263	0.153	0.066
Layer thickness (mm)	0.280			
Printing speed (mm/s)	0.800			

**Table 2:** Elastic modulus and strain at break with respect to dye concentration.

Concentration (wt.%)	Elastic Modulus (MPa) *	Strain at Break *
0	176.778 ± 5.805	0.198 ± 0.038
5	235.308 ± 4.274	0.235 ± 0.036
10	255.618 ± 9.223	0.201 ± 0.061
15	325.335 ± 12.954	0.208 ± 0.030

* Average ± standard deviation.

**Table 3:** Color brightness of the membranes at room temperature and 37.5 °C and time constant during thermochromic reaction.

Concentration (wt.%)	Brightness *		Time Constant (s) *
	At Room Temp.	At 37.5 °C	
5	86.835 ± 3.231	141.027 ± 2.549	0.195 ± 0.036
10	74.647 ± 2.896	135.237 ± 3.310	0.395 ± 0.088
15	65.733 ± 4.435	128.955 ± 1.292	0.552 ± 0.077

* Average ± standard deviation.

**Table 4:** Printing conditions for skin patch with lattice-pattern.

Parameter (Unit)	Value
Concentration (wt.%)	5
Barrel temperature (°C)	100
Ambient temperature (°C)	20
Pressure (kPa)	600
Nozzle inner diameter (mm)	0.130
Tip to collector distance (mm)	0.120
Collector feedrate (mm/s)	0.500
Pitch (mm)	0.250

## Data Availability

Data sharing not applicable.

## Supplementary Materials

The following are available online at https://www.mdpi.com/article/10.3390/ma14133460/s1, Figure S1: The behavior of R, G, B, and brightness value when a patch had color change due to thermochromic reaction. Figure S2: Cross-sections of the printed filament (pigment concentration: 0 wt.%). Figure S3. RGB value investigation results for 15 points. Table S1. Statistical information of RGB value investigation results for 15 points.
