# Supplementary material for: Fabrication of Thermochromic Membrane and Its Characteristics for Fever Detection
Source: Materials (Basel). 2021 Jun 22;14(13):3460. doi: 10.3390/ma14133460 (PMC8269493; doi:10.3390/ma14133460)
Supplement: Supplementary file 1 [file materials-14-03460-s001.zip › materials-1232097-supplementary.pdf]

materials

Supplemetary Materials: Fabrication of Thermochromic Membrane and Its Characteristics for Fever Detection

Hyeon Seop Jeon [1], Jeong Hwa Kim [1], Martin B. G. Jun [2] and Young Hun Jeong [3,*]

[1] Department of Mechanical Engineering, Graduate School, Kyungpook National University, Daegu 41566, Korea; hsjeon1994@gmail.com (H.S.J.); qhfekrn89@gmail.com (J.H.K.)
[2] School of Mechanical Engineering, Purdue University, West Lafayette, IN 47907, USA; mbgjun@purdue.edu
[3] School of Mechanical Engineering, Kyungpook National University, Daegu 41566, Korea
* Correspondence: yhjeong@knu.ac.kr

Citation: Jeon, H.S.; Kim, J.H.; Jun, M.B.G.; Jeong, Y.H. Fabrication of Thermochromic Membrane and Its Characteristics for Fever Detection. *Materials* **2021**, *14*, 3460. https://doi.org/10.3390/ma14133460

Received: 07 May 2021
Accepted: 15 June 2021
Published: 22 June 2021

Publisher's Note: MDPI stays neutral with regard to jurisdictional claims in published maps and institutional affiliations.

Copyright: © 2021 by the authors. Licensee MDPI, Basel, Switzerland. This article is an open access article distributed under the terms and conditions of the Creative Commons Attribution (CC BY) license (http://creativecommons.org/licenses/by/4.0/).

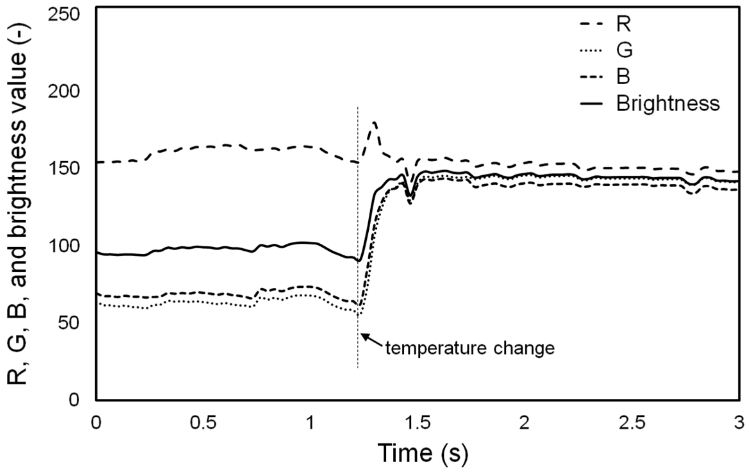

Figure S1. The behavior of R, G, B, and brightness value when a patch had color change due to thermochromic reaction.

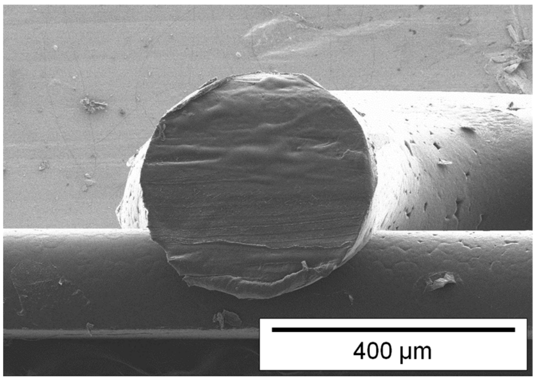

(**a**) at the middle of overhanging region

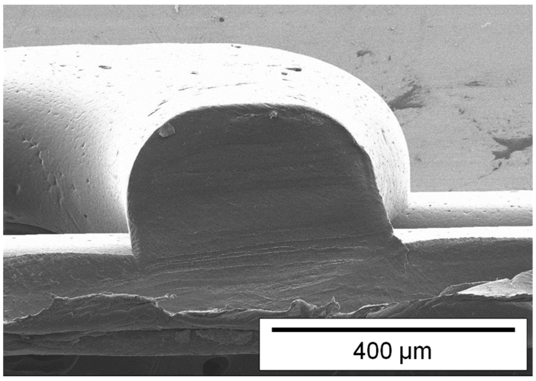

(**b**) at the layer intersection region

Figure S2. Cross-sections of the printed filament (pigment concentration: 0 wt.%).

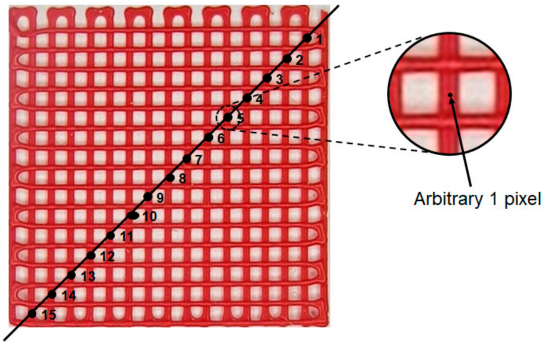

(**a**) check spots

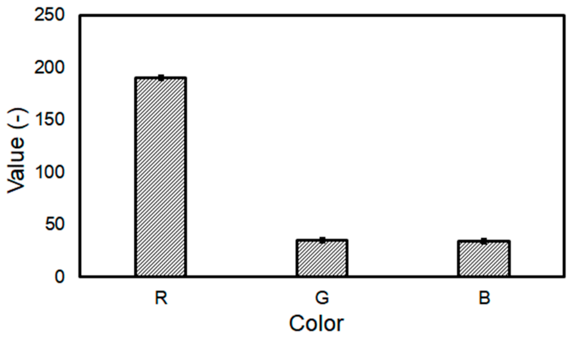

(**b**) RGB values

Figure S3. RGB value investigation results for 15 points.

Table S1. Statistical information of RGB value investigation results for 15 points.

Color	R	G	B
Value*	190.333 ± 2.316	35.310 ± 2.483	34.286 ± 2.281

* average ± standard deviation.
